# Ethephon as a bloom-delaying agent for frost-sensitive tree crop flowers at risk from a changing climate

**DOI:** 10.1186/s12870-026-09346-4

**Published:** 2026-07-09

**Authors:** Anna Maria Geisthoff, Marco Ferrante, Wiebke Kämper

**Affiliations:** Agroecology & Functional Agrobiodiversity, Department of Crop Sciences, University of Göttingen, Göttingen, 37077 Germany

**Keywords:** Crop production, Delayed flowering, Frost damage, Ethylene, *Malus domestica*, *Prunus avium*, Phenology, Spring frost

## Abstract

**Background:**

Climate change increases the risk of late spring frosts, which damage flowers and developing fruitlets in tree crops. Ethephon, a plant growth regulator, may delay flowering and potentially reduce frost damage.

**Methods:**

We investigated the effects of ethephon on flowering time and fruit set in two sweet cherry (Bellise and Kordia) and two apple cultivars (Elstar and Kanzi). Treatments included two ethephon concentrations (500 and 750 ppm) applied either in early or late autumn.

**Results:**

Ethephon significantly delayed flowering in cherry cultivar Kordia, but not cultivar Bellise, with a lower proportion of Kordia floral buds in bloom in two of four treatments compared to controls. Specifically, on 9 April 88% of floral buds on control trees were in bloom, compared to 56% and 63% on trees treated with high ethephon in early autumn and low ethephon in late autumn, respectively. No significant flowering delay was observed in the apple cultivars. Initial and final fruit set were generally unaffected, although Kordia showed a reduction in final fruit set from 16 to 6% after the late-high treatment.

**Conclusions:**

Our results indicate that ethephon can effectively delay bloom without major yield loss, offering a practical tool to mitigate spring frost damage under climate change. However, its effectiveness was crop- and cultivar-dependent.

**Supplementary Information:**

The online version contains supplementary material available at https://doi.org/10.1186/s12870-026-09346-4.

## Introduction

Fruit production plays a vital role in global nutrition [15]. Fruits provide essential nutrients such as vitamin C, A- and B-vitamins, and minerals like potassium and magnesium [48]. The high fibre and antioxidant contents of fruits also help prevent cardiovascular diseases and cancer, which cause millions of deaths every year [10, 56]. Global fruit and vegetable production must increase by 50 to 150% by 2050 to meet the dietary needs of 10 billion people [14, 58]. However, climate change is directly threatening crop production and food security, with extreme weather events and changes in abiotic conditions aggravating production losses [2]. Climate change has shifted flowering of many crops to earlier in the year, exposing flowering crops to more erratic temperatures including spring frosts [38, 39]. In temperate regions, climate change strongly affects the occurrence and the intensity of late frost events, and models predict these events could intensify by about 30% during the growing period of fruit trees if global temperatures rise by 2 °C [53].

Climate change may cause warmer spring temperatures, altering plant phenology and increasing the severity of damage caused by spring frost events. Temperate plants are in sensitive phenological stages after breaking dormancy and during bud break [4]. The earlier bud break of many temperate plants leads to a prolonged overlap with spring frost events, so that damage to crop production and economic losses become increasingly likely [28]. A meta-analysis found that between 1971 and 2000, leafing, flowering, and fruiting events occurred significantly earlier in 30% of 542 plant species, strongly correlating with temperature changes [38]. Shorter dormancy periods and earlier bud bursts and flowering have been reported for many woody perennials across the Northern Hemisphere [4, 34]. An especially severe frost event took place in Europe in April 2017 [55]. Across Europe, the damage in fruit orchards and vineyards was estimated as 3.3 billion Euros [17]. Similarly, in the United States, a frost event in April 2007 resulted in losses of approximately 2 billion US Dollars [42].

Different approaches to mitigate the effects of frost for flowers and fruit on trees exist. Active methods include the modification of the orchard microclimate using wind machines, surface irrigation systems, and heaters [32]. While these techniques have immediate and direct effects, they are typically cost-inefficient and environmentally unsustainable. Passive strategies are mainly preventive, and include the cultivation of cold-tolerant or late-flowering cultivars and careful site selection prior to plantation establishment [32, 49]. Plant growth regulators have also been used to improve freeze tolerance [11, 43] or to delay flowering, hereby evading potential frost damage [20, 40]. Main advantages of plant growth regulators are their affordability and the ease of implementation [32].

Ethephon is a plant growth regulator that affects plant development by conversion into ethylene upon entering the cytoplasm [45]. It is widely used for agricultural purposes. For example, to speed up ripening in tomatoes [6], or to inhibit growth and avoid lodging in grain [45]. Ethephon is also used for flower and fruit thinning in many fruit and nut trees such as apple, macadamia, olive and peach [37, 50, 51], hereby reducing alternate bearing [37, 46]. Ethephon has also been shown to delay flowering in crops such as pistachio, blueberry, and some stone fruits such as almond, apricot, peach, and plum by increasing chilling requirements and extending dormancy [3, 7, 13, 20, 26, 31, 32, 40]. The effect of ethephon on temperate crops, particularly commercially important pome fruits, has received little to no attention, despite their vulnerability to late spring frost damage [52]. Ethephon can also have adverse effects on tree health, causing stress responses in dormant buds [31], and increasing the occurrence of gummosis in, for example, some stone fruits [20, 29]. Additionally, ethephon use can reduce the number of flower buds and increase flower and fruit abscission [26, 32]. However, results have been inconsistent, even within the same crop plant. Potential adverse effects, along with limited knowledge on how to apply ethephon correctly and most efficiently, can hinder its commercial use, so additional research is needed.

This study aims to determine the effect of ethephon application on the timing of flowering and on initial and final fruit set in two sweet cherry and two apple cultivars. Cherries and apples are significant crops within the EU. In 2023, 566 thousand tons of cherries were produced in the EU, equating to more than 1 kg per capita, whereas 12.1 million tons of apples were produced, equating to nearly 27 kg per capita [16]. These crops were also chosen because promising results for flowering delay have been found in other stone fruits and because, to our knowledge, only a single study has investigated the potential effects on pome fruits despite their immense economic value. Two concentrations of ethephon were applied at either early autumn, when trees had dropped about 10% of leaves, or at late autumn, when trees had dropped about 50% of leaves. Our objectives were to (i) investigate whether ethephon could delay flowering by comparing the proportion of floral buds in bloom between treated trees and control trees on multiple days during peak flowering, and (ii) compare the fruit set (initial and final) between treated trees and control trees. We predicted that ethephon delays flowering, with the higher concentration causing a pronounced delay compared to the control trees. We furthermore predicted a reduced fruit set in treated trees compared to control trees at the higher concentration.

## Materials and methods

### Study sites and cultivar information

The study was conducted in Central Germany, near Stadthagen, in one sweet cherry orchard (52.3278, 9.2553) and one apple orchard (52.3248, 9.2354). The sweet cherry (*Prunus avium*) orchard covered 1.1 ha with multiple cultivars planted mostly in single rows. The soil was loamy loess (Geodata of the Lower Saxony Soil Information System (NIBIS)). We chose Bellise and Kordia as experimental cultivars. Bellise is early flowering and Kordia has particularly frost-sensitive flowers despite being medium-late flowering, so that both cultivars are experiencing a high frost damage risk. Kordia is also one of the most important sweet cherry cultivars in Europe including Germany (personal communication; Vávra et al. [54]. Bellise and Kordia trees were planted in 2008 and the tree spacing was 4.5 m between rows. Tree spacing within the row was 2.5 m for Bellise, and 3 m for Kordia. The set-up of the sweet cherry orchard mostly followed the alternate row design, where trees of one cultivar are planted in a single row next to a single row of another cultivar. The experimental trees experienced open pollination. Managed pollinators were introduced by the farmer during flowering, installing three honeybee hives and 12 commercial bumblebee (*Bombus terrestris*) hives.

The apple (*Malus domestica*) orchard covered 2.7 ha with multiple cultivars planted in single rows. The soil was also loamy loess (NIBIS). We chose Elstar and Kanzi as experimental cultivars because both cultivars have especially frost-sensitive flowers. Additionally, Elstar is one of the most popular apple varieties in Germany, having a share of 32% of apple production area in Lower Saxony in 2019 [41]. Elstar and Kanzi trees were planted in 2011 and the tree spacing was 3.5 m between rows and 1 m within rows for both cultivars. The set-up of the apple orchard followed the alternate row design, where trees of one cultivar are planted in a single row next to a single row of another cultivar. Often every tenth tree in a row was a crab apple tree that provides an alternative pollen genotype. The experimental trees experienced open pollination.

Chilling requirements are cultivar specific, and describe how many chill hours (often between 0–7 °C) a deciduous tree must experience in winter to exit endodormancy and commence bud break. Kordia and Bellise are high chill sweet cherry cultivars, with estimates around 1,000–1,500 chill hours required [30, 36]. Similarly, Kanzi and Elstar are high-chill apple cultivars, with estimates above 1,000 chill hours required [8]. The daily average temperature at our study site across the three winter months (December 2023, January 2024, February 2024) was 5.3 °C, the daily average minimum temperature was 1.5 °C and the cumulative precipitation 305 mm during the experimental winter. During flowering, in April 2024, the daily average temperature was 11.1 °C, the daily average minimum temperature was 5.4 °C and the precipitation 56 mm.

### Experimental design

**Fig. 1:**
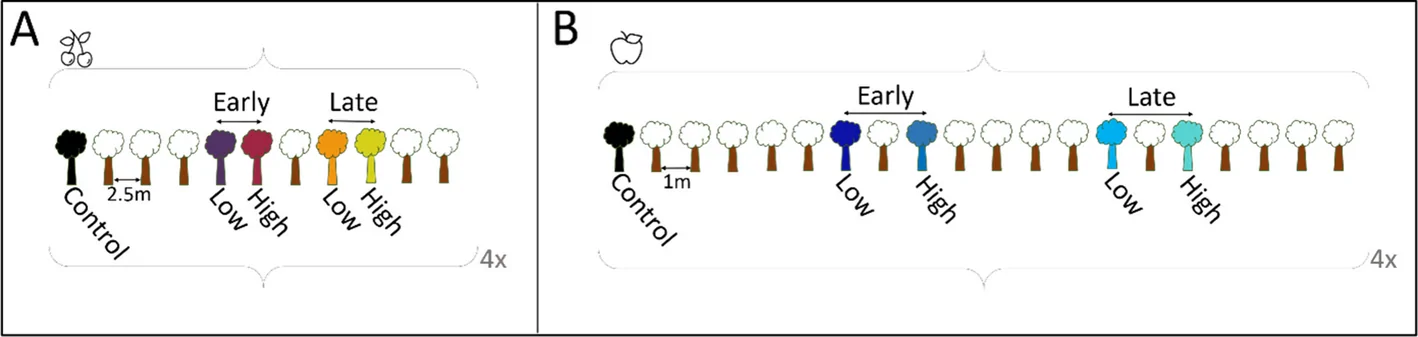
One of four experimental units in one (**A**) sweet cherry and one (**B**) apple cultivar. Black trees represent control trees, and coloured trees represent experimental trees that received an ethephon treatment in early or late autumn at either a low or high dose. White trees represent buffer trees. More buffer trees were included in apple to avoid ethephon drift to neighbouring trees given the much smaller tree spacing in apple (1 m vs. 2.5 m)

We selected 20 experimental trees per sweet cherry and apple cultivar, with four control trees and four trees assigned to each of four treatments. An ethephon solution (Cerone 660; Bayer, Leverkusen, Germany) was directly applied until run-off to the treatment trees using a backpack sprayer with double cone nozzles during dry and windless weather conditions. Experimental trees were assigned to one of five treatments: (a) control trees; (b) 500 ppm ethephon, application at 10% leaf-fall; (c) 750 ppm ethephon, application at 10% leaf-fall; (d) 500 ppm ethephon, application at 50% leaf-fall; (e) 750 ppm ethephon, application at 50% leaf-fall. Early and late application in sweet cherry took place on 16 October and 16 November 2023, respectively, whereas early and late application in apple took place on 17 November and 7 December 2023, respectively. Hereafter, the treatments are called early-low, early-high, late-low, and late-high, respectively. Concentrations were chosen based on previous studies investigating the beneficial and adverse effects of autumn-applied ethephon in fruit trees and bushes [32]. Application time points were chosen because the highest effectiveness for bloom delay is expected early, when floral buds are less advanced and more responsive. However, at 10% leaf-fall most leaves are present, enabling a higher ethephon uptake by the tree, increasing the risk of adverse effects. Experimental trees per cultivar were located within one row, and arranged in experimental units (Fig. 1) that included buffer trees to prevent that spray drift affected the results. The distance from the first experimental trees to the edge of the orchard measured at least 12 m. All four experimental units per cultivar were adjacent to each other, and each experimental unit in sweet cherry was 27.5 m long, whereas experimental units in apple were 20 m long.

### Data collection

We tagged four branches per experimental tree, with one in each cardinal direction, and recorded the total number of flower buds per branch, focusing on the area between the tag and the tip of the branch. Branch sections had on average (± SE) 30 ± 0.4 floral buds for cherry and 15 ± 0.2 floral buds for apple. For both cherry and both apple cultivars, each flower bud was counted and assigned to its phenological stage during seven visits during flowering between 1 and 30 April 2024. These visits were used to calculate the proportion of flower buds in bloom, as a measure for timing of flowering. Another two visits per cultivar were conducted, with one for both crops taking place on 16 May 2024 to record initial fruit set and one to record final fruit set. Final cherry fruit set was recorded shortly before harvest on 7 June 2024 for Bellise and 13 June 2024 for Kordia. Final apple fruit set was recorded after June drop on 19 July 2024, as this represents the fruits that most likely will remain until harvest.

### Data analysis

We used the R packages glmmTMB for fitting the GLMMs, DHARMa for simulation-based residual diagnostics, ggeffects for visualisations, and lsmeans for pairwise comparisons [22,27, 33, 35]. Means are reported with standard errors. Details of individual models and the R code are presented in the supplementary material.

### Analysis of flowering delay

For each cherry and apple cultivar, we tested whether the interaction of treatment and date affected the proportions of floral buds in bloom using a generalised linear mixed model (GLMM) with a beta distribution and logit link function. Tree ID was included as a random effect. To account for zero inflation, the ziformula incorporated treatment, date, and tree ID. To address heteroscedasticity, the dispformula included tree ID. Dates with fewer than 4% flowering floral buds were excluded from the analysis to improve the robustness and fit of the statistical models. These dates provided limited information and introduced substantial zero inflation. The final models for the sweet cherry cultivar Kordia exhibited minor underdispersion and heteroscedasticity, while the final models for both apple cultivars showed minor underdispersion.

###  Analysis of fruit set

For each cherry cultivar, we tested whether treatment affected the proportion of floral buds that initially set fruit, and the proportion of floral buds that resulted in cherry fruit retained until harvest using a zero-inflated GLMM (i.e., ziformula = ~ 1) with a beta distribution and logit link function. Tree ID was included as a random effect. For each apple cultivar, we tested whether treatment affected the proportion of floral buds that initially set fruit, and the proportion of floral buds that resulted in a fruit retained until after the June drop event, using a GLMM with Gaussian distribution and identity link function. We included tree ID as a random effect. For cultivar Elstar, we, added tree ID to the dispformula to address heteroscedasticity.

## Results

### Flowering delay in sweet cherry

**Fig. 2:**
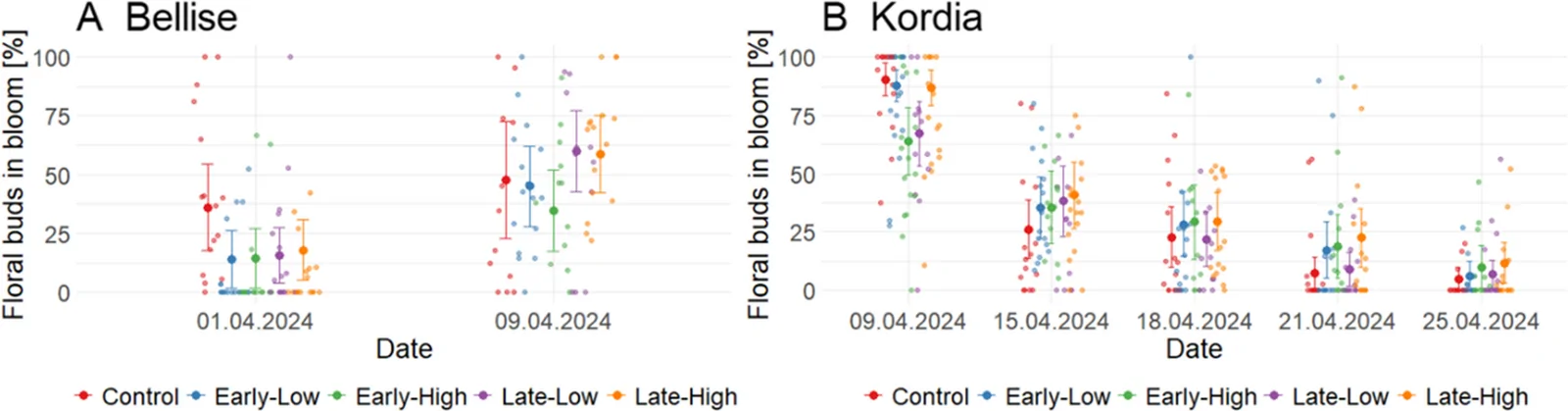
Percentage of floral buds in bloom and predicted percentage of floral buds in bloom on untreated control trees and on ethephon-treated trees in the sweet cherry cultivars (**A**) Bellise and (**B**) Kordia. Means (± SE) between treated and control trees within each date for the proportion of floral buds in bloom were generally not significantly different (LSmeans *p* > 0.05), except for a significantly lower proportion of floral buds in bloom on trees that received the early-high and late-low treatment compared to control trees in cultivar Kordia on 9 April 2024 (LSmeans *p* < 0.05, *n* = 4 trees per treatment)

Flowering could not be delayed in Bellise, with proportions of floral buds in bloom not significantly differing between treated and control trees on 1 and 9 April 2024 (Fig. 2A; Table S1 for statistical output). On 1 April, however, the proportion of floral buds in bloom was 42 ± 9% on the control trees in comparison to 11 ± 4% in the early-low, 9 ± 6% in the early-high, 18 ± 7% in the late-low, and 9 ± 3% in the late-high treated trees, respectively. Values were much more similar across treatments on 9 April, with 38 ± 11% on the control trees in comparison to 44 ± 7% in the early-low, 40 ± 8% in the early-high, 51 ± 10% in the late-low, and 63 ± 6% in the late-high treated trees, respectively.

In contrast, flowering in cultivar Kordia could be delayed by the early-high and late-low treatments compared to the control. Specifically, on 9 April the proportion of floral buds in bloom was significantly lower (LSmeans, estimate = 2.21, SE = 0.48, *p* < 0.01; estimate = 1.99, SE = 0.49, *p* = 0.01) on the early-high (56 ± 7%) and on the late-low treated trees (63 ± 8%) compared to the control trees (88 ± 5%). The other treatments did not change the proportion of floral buds in bloom compared to the control trees (Fig. 2B; Table S2). Similarly, on 15, 18, 21, and 25 April 2024, the proportion of floral buds in bloom in Kordia did not significantly differ between any treatment compared to the control trees (Table S2).

### Flowering delay in apple

**Fig. 3:**
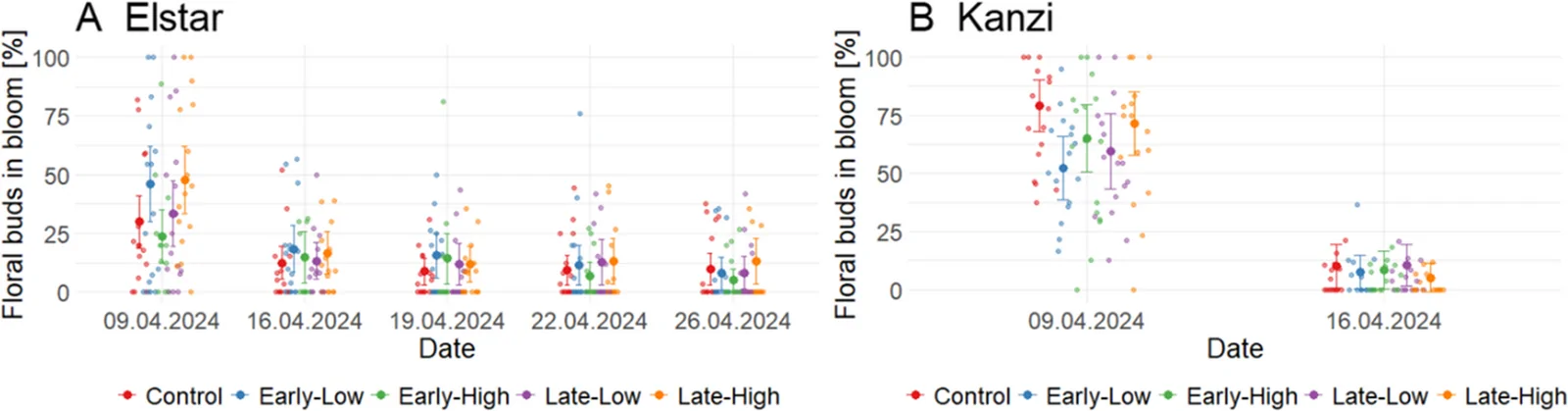
Percentage of floral buds in bloom and predicted percentage of floral buds in bloom on untreated control trees and on ethephon-treated trees in the apple cultivars (**A**) Elstar and (**B**) Kanzi. Means (± SE) between treated and control trees within each date for the proportion of floral buds in bloom were not significantly different (LSmeans *p* > 0.05; *n* = 4 trees per treatment)

Flowering could not be delayed in Elstar or Kanzi, with proportions of floral buds in bloom not significantly differing between treated trees and control trees (Fig. 3; Tables S3-S4). On 9 April in cultivar Kanzi, however, the proportion of floral buds in bloom was 73 ± 5% on the control trees in comparison to 54 ± 5% in the early-low, 59 ± 8% in the early-high, 55 ± 6% in the late-low, and 65 ± 7% in the late-high treated trees, respectively. Values were more similar across treatments on 16 April, with 5 ± 2% on the control trees in comparison to 5 ± 2% in the early-low, 5 ± 1% in the early-high, 5 ± 2% in the late-low, and 2 ± 1% in the late-high treated trees, respectively.

### Fruit set in sweet cherry

**Fig. 4:**
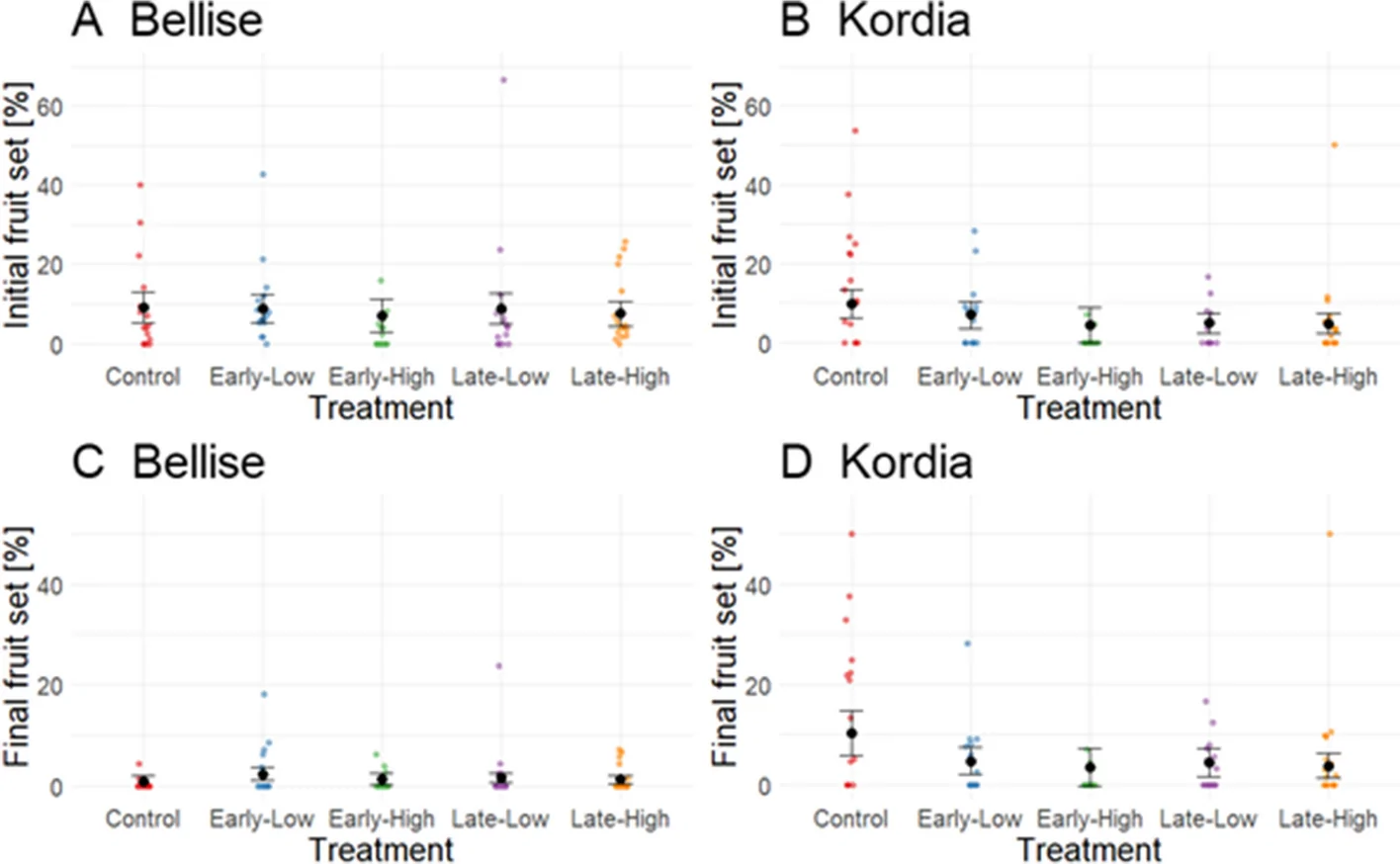
Initial fruit set and predicted initial fruit set on 16 May 2024 in sweet cherry cultivars (**A**) Bellise and (**B**) Kordia, and final fruit set (pre-harvest) and predicted final fruit set (**C**) on 7 June 2024 in cultivar Bellise and (**D**) on 13 June 2024 in cultivar Kordia on untreated control trees and on ethephon-treated trees. Means (± SE) between treated and control trees were generally not significantly different (LSmeans *p* > 0.05), except for a significantly lower final fruit set on trees that received the late-high treatment compared to control trees in cultivar Kordia (LSmeans *p* < 0.05; *n* = 4 trees per treatment)

The average initial fruit sets were 8 ± 1% and 7 ± 1% for Bellise and Kordia, respectively, whereas the final fruit sets were 1.5 ± 0.5% and 6 ± 1% for Bellise and Kordia, respectively. Initial fruit set did not differ between treatment trees and control trees in either Bellise or Kordia (Fig. 4A-B; Tables S5-S6). Final fruit set, however, was lower on late-high treated trees compared to the control trees in cultivar Kordia (LSmeans, estimate = 1.16, SE = 0.42, p = 0.049), whereas final fruit set in cultivar Bellise did not differ between treated and control trees (Fig. 4C-D; Tables S7-S8).

### Fruit set in apple

**Fig. 5:**
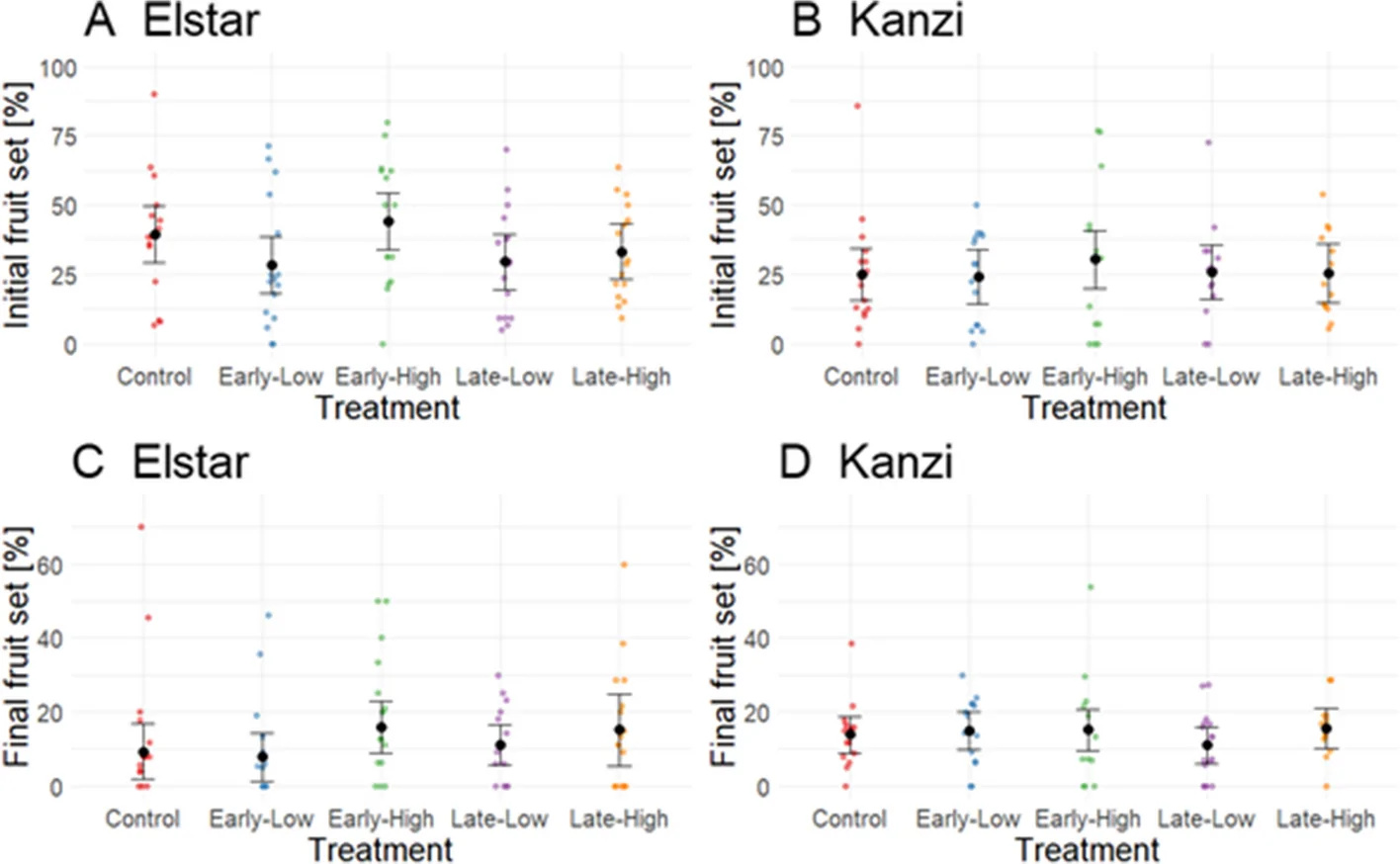
Initial fruit set on 16 May 2024 in apple cultivars (**A**) Elstar and (**B**) Kanzi, and final fruit set on 19 July 2024 (after June fall) in apple cultivars (**C**) Elstar and (**D**) Kanzi on untreated control trees and on ethephon-treated trees. Means (± SE) between treated and control trees were not significantly different (LSmeans, *p* > 0.05; *n* = 4 trees per treatment)

The average initial fruit sets were 35 ± 5% and 26 ± 4% for Elstar and Kanzi, respectively, whereas the final fruit sets were 15 ± 4% and 14 ± 2% for Elstar and Kanzi, respectively. Neither initial nor final fruit set differed between treated trees and control trees in either Elstar or Kanzi (Fig. 5 A-D; Tables S9-S12).

## Discussion

In this study, we assessed the effectiveness of ethephon applications, administered in early or late autumn, in delaying flowering in two sweet cherry cultivars and two apple cultivars. Our results demonstrate that ethephon can successfully postpone flowering in some cases. Specifically, in the sweet cherry cultivar Kordia, flowering was significantly delayed on 9 April, where control trees exhibited more advanced flowering compared to those treated with a high dose of ethephon in early autumn or a low dose in late autumn. In contrast, flowering delay was not observed in the sweet cherry cultivar Bellise or both apple cultivars. Across both sweet cherry and apple cultivars, ethephon treatments generally had minimal adverse effects on fruit set, with initial and final fruit set remaining comparable between treated and control trees. Our findings indicate that ethephon application in autumn can offer a promising alternative to labour-intensive frost mitigation strategies for some sweet cherry cultivars. By delaying flowering, ethephon may help protect flowers and developing fruitlets from spring frost damage, which becomes increasingly important and urgent given the rising incidence of late spring frosts associated with climate change.

Our findings demonstrate that ethephon delayed flowering in the sweet cherry cultivar Kordia, but not in the sweet cherry cultivar Bellise or either apple cultivar, showing how the effects of ethephon application were cultivar-dependent, and crop- and treatment-specific. We observed that both the early-autumn high-dose and the late-autumn low-dose applications resulted in significantly delayed flowering compared to the control in the sweet cherry cultivar Kordia on 9 April. Studies on other stone fruit such as apricot, peach, and plum showed that ethephon can be used to delay flowering from a couple of days up to two weeks [7, 13, 20, 26, 31, 32, 40]. To our knowledge only one study tested ethephon in concentrations of 250 to 500 ppm to delay flowering in apples, but, similar to our results, found no effect [9]. Studies on apple have used ethephon for flower and fruit thinning, which improves return bloom and ultimately reduces alternate bearing, a phenomenon where trees produce abundant fruits in one year and very few flowers and fruits in the following year [12, 21, 23, 24, 46]. This shows that phenological development of apple trees can be influenced by application of ethephon, although an effect on flowering time could not be detected in this study. Apple flowering did, however, start exceptionally early in 2024. In northern Germany, Elstar flowered earlier than ever recorded, with flowering beginning 17 days earlier than the long-term mean [57], which may have shortened and intensified bloom during our study period, possibly further impeding the observation of differences between treatments.

In Kordia, a significant bloom delay could be observed when trees were treated with a high dose of ethephon early, at 10% leaf-fall, or with a low dose late, at 50% leaf-fall. Generally, research indicates that a higher concentration of ethephon and earlier application in autumn leads to a stronger bloom delay [32]: This is also supported by research on two stone fruits, peach and prune, finding that the effect of ethephon on bloom delay was higher at 10% leaf-fall compared to 50% leaf-fall [7]. Leaves are the main part of the plant through which ethephon can enter the cytosol and convert into ethylene [18, 44, 45]. Consequently, an increase in leaf surface area would lead to higher uptake of ethephon, resulting in greater ethylene production. Additionally, floral buds are in less advanced stages at the earlier application time, and these younger floral buds might be more responsive than floral buds that are further along in their development [7]. Regardless, our results show that not only the high dose treatment in early autumn but also the low dose treatment in late autumn can initiate flowering delay. The observation that the late-low ethephon treatment delayed bloom in Kordia, while the late-high treatment did not, suggests that the relationship between dose, timing, and physiological response are non-linear. For example, high concentrations of ethephon applied late in the season may induce excessive ethylene production, leading to phytotoxic effects such as leaf abscission, gummosis, or even premature bud desiccation. Such stress responses can disrupt normal dormancy progression and may actually reduce the efficacy of bloom delay, as the plant prioritises stress recovery over dormancy modulation. In contrast, a lower dose at the same timing may provide a subtler ethylene signal that is sufficient to modulate bud dormancy and increase chilling or heat requirements, without triggering damaging side effects.

Generally, we found minimal adverse effects on initial or final fruit set with ethephon treatment for either sweet cherry or apple cultivar. The only effect on fruit set we found was in sweet cherry cultivar Kordia, where trees receiving the late-high ethephon treatment had a reduced final fruit set of 6% compared to 16% fruit set in the control trees. This could be a result of excessive ethylene production after high concentration of ethephon applied late in the season. Previous studies on various tree and shrub crops have provided inconclusive results. For example, ethephon application of up to 500 ppm has been shown to reduce yields in almond, blueberry, and peach [7, 20, 26], whereas an increase in yield following 500 ppm ethephon application was reported for sweet cherry [47]. In apple, ethephon applied after full bloom has been reported to enhance yield in the following year [46]. In contrast, a reduction of 83 to 100% in apple fruit set was observed following autumn application of 250 to 1000 ppm [25]. The mixed results suggest that the effect of ethephon may depend on many factors such as species identity, cultivar, tree age, growing conditions, and region, and specifics of the application such as the concentration and timing. From our results, we would recommend to apply ethephon earlier because the risk of adverse effects may be lower at 10% leaf-fall.

Individual sweet cherry flowers are flowering for 2–5 days, during which they also produce nectar [1, 5]. However, flowering can take anywhere from a couple of days to more than two weeks, when looking at the tree level [5]. The flowering period of sweet cherry trees was extremely early and short in 2024. In northern Germany, cultivar Regina started to bloom on 8 April, much earlier than the long-term average bloom date of 20 April (1979 to 2023) [57]. The unusually warm weather in the first half of April led to twelve cultivars at our study site having passed their peak flowering by 15 April, with only one cultivar, Regina, yet to reach peak flowering. The exceptional short and early flowering may have reduced the window for observing differences between treated and control trees, and the bloom-delaying effect of ethephon might be even more pronounced under typical climatic conditions. We recognise that our findings may not be generalisable to other cultivars, growing regions, or years with more typical phenological conditions. Thus, studying the effects of ethephon across many cultivars, multiple orchards, and years, and incorporating assessments of tree physiology, would enhance our understanding on to what extent ethephon consistently causes flowering delay in certain cultivars, and reveal underlying physiological mechanisms. Another limitation of our study is the admittedly small sample size per treatment. There are indications that flowering delay may also have occurred in the sweet cherry cultivar Bellise, particularly on 1 April, and the apple cultivar Kanzi, particularly on 9 April, where flowering of control trees might have been more advanced compared to the treated trees. The exceptionally early and condensed flowering period in 2024, driven by unseasonably warm weather, and the small sample size per treatment likely influenced our ability to detect effects. The complexity of ethephon effects warrant further research to investigate the effects on different crops and cultivars, especially given that we might have observed a non-linear response to ethephon application in Kordia. Investigating the potential of ethephon to increase cold hardiness in dormant buds is also warranted. For example, in peach it has been reported that 30 to 40% of buds treated with ethephon stood through temperatures below –20 °C, whereas only about 10% of the untreated buds did [19].

In conclusion, our study demonstrates that ethephon treatment can effectively delay flowering in the sweet cherry cultivar Kordia without negatively impacting fruit set. Understanding the specifics and dynamics of ethephon application is crucial as climate change continues to impact fruit production, urging the need for adaptive strategies to secure future yields and food stability while spring frost events become increasingly common. Overall, this study contributes insights into the potential of effective frost mitigation through autumn-applied ethephon instead of traditional, labour-intensive frost mitigation measures such as wind machines, heaters, or overhead irrigation. Its role in mitigating spring frost risk is likely to become increasingly important as climate change challenges traditional orchard management practices.

## Supplementary Information

Supplementary Material 1.

## Data Availability

Data and R scripts are available under DOI: https://doi.org/10.25625/VRG9NG.
